# Dermatopathia pigmentosa reticularis and overlap syndrome in siblings—A rare case report

**DOI:** 10.1016/j.jdcr.2026.04.062

**Published:** 2026-05-07

**Authors:** Midhun Raj, Vimal Thomas, Tarun Kumar Suvvari, Anand Krishnan RG, Annmariya Joseph, Ansila Anna Jaimon, Aasim Akthar Ahmed

**Affiliations:** a General Hospital, Trivandrum, Kerala, India; b Tbilisi State Medical University, Tbilisi, Georgia; c Rare Disease Research Group (RDRG), Squad Medicine and Research (SMR), Amadalavalasa, Andhra Pradesh, India; d Rangaraya Medical College, Kakinada, Andhra Pradesh, India; e Government Medical College, Thiruvananthapuram, Kerala, India; f David Tvildiani Medical University, Tbilisi, Georgia; g St. Francis Medical Center, Monroe, Louisiana

**Keywords:** dermatopathia pigmentosa reticularis, familial skin disorder, Naegeli–Franceschetti–Jadassohn, overlap syndrome

## Introduction

Naegeli–Franceschetti–Jadassohn (NFJ) syndrome is a rare form of ectodermal dysplasia affecting skin, sweat glands, nails, and teeth.^1^ Dermatopathia Pigmentosa Reticularis (DPR) is another autosomal dominant ectodermal dysplasia syndrome that clinically shares features with NFJS, such as the complete absence of dermatoglyphics, a reticulate pattern of skin hyperpigmentation, palmoplantar keratoderma, abnormal sweating, and various subtle developmental anomalies of the teeth, hair, and skin.^2^ It is also significant to note that both NFJS and DPR exhibit distinct clusters of symptoms indicating differences across genotypes and phenotypes.^3^

We report a rare case involving 2 siblings: case 1, a 24-year-old male with a diagnosis of DPR, and case 2, his 28-year-old sister demonstrating features of an overlap ectodermal dysplasia syndrome.

## Case 1

A 24-year-old male arrived at the outpatient clinic with symptoms of diffuse brown hyperpigmentation, notable scalp hair thinning, and discoloration along with fragile fingernails and toenails. The hyperpigmentation was first observed at birth, initially confined to the trunk, but progressively spread over the next 3 years to include the proximal limbs, palms, soles, head, neck, and tongue within that period. The thinning of the scalp hair and nail issues began with the onset of puberty. Along with the skin and nail changes, he also exhibited a lack of dermatoglyphics, excessive sweating, and an intolerance to heat. The patient reported no prior injuries to the fingers or exposure to chemicals.

**Fig 1:**
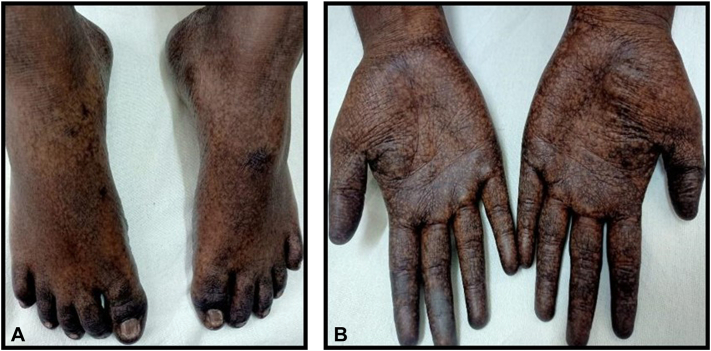
Reticulate hyperpigmentation of the proximal limbs. **A,** feet. **B,** hands.

**Fig 2:**
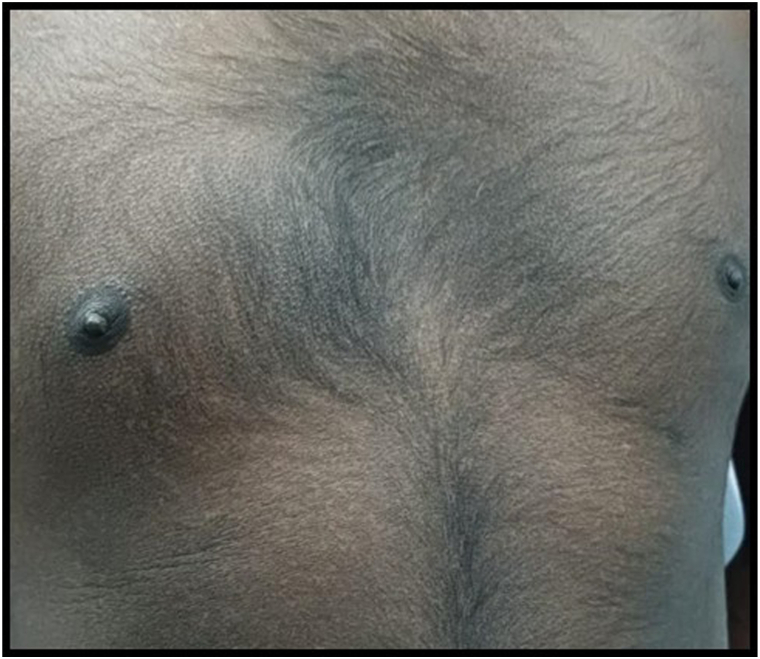
Increased pigmentation of areola.

**Fig 3:**
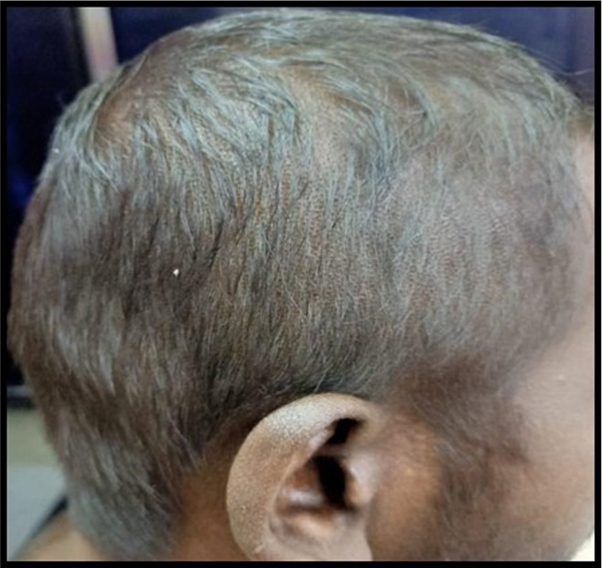
Diffuse, nonscarring hair loss of the scalp.

**Fig 4:**
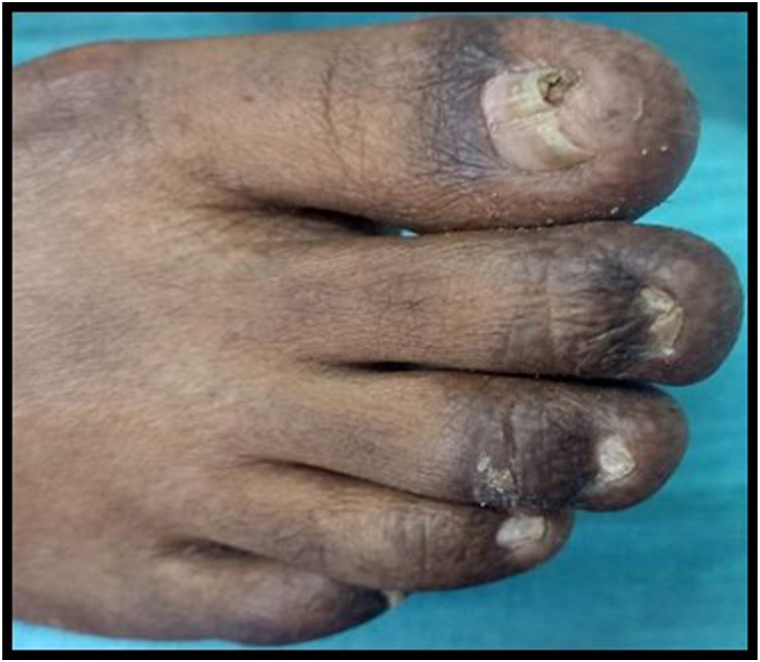
Onychodystrophy of toenails.

**Fig 5:**
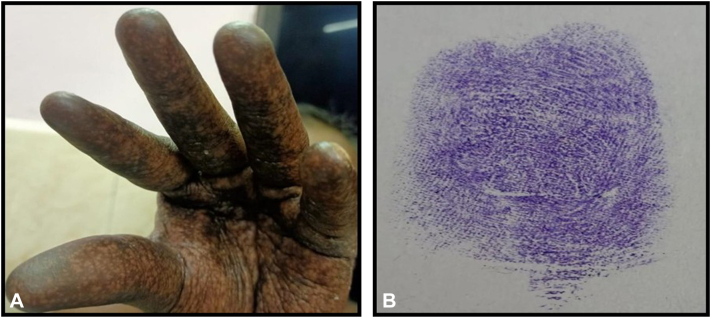
Absent dermatoglyphics.

**Fig 6:**
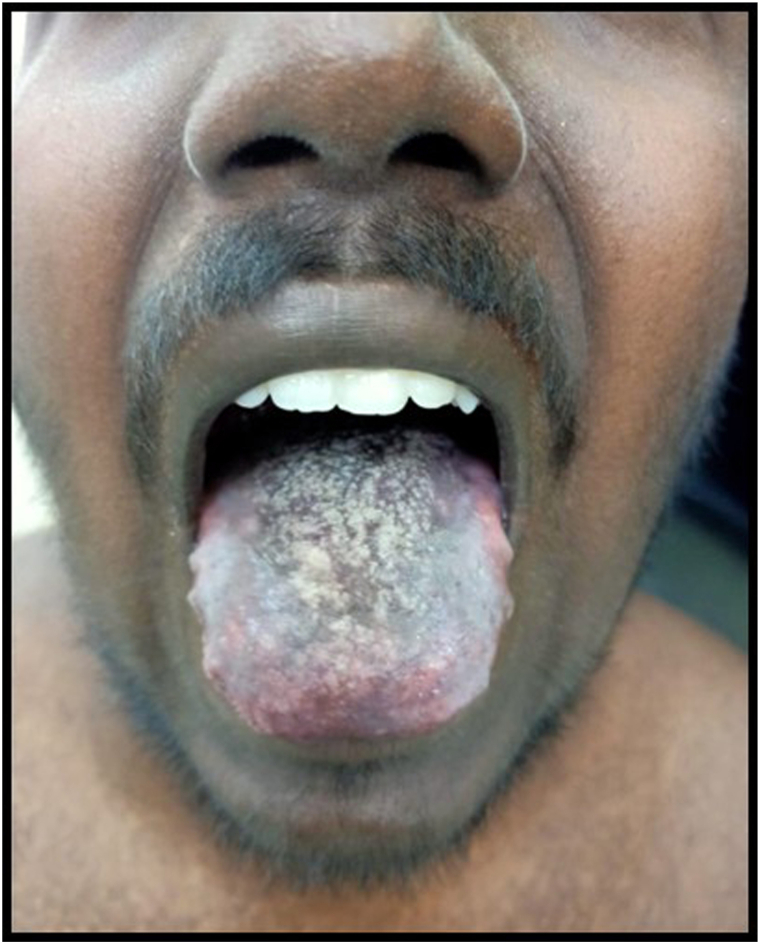
Mucosal lesions and diffuse hyperpigmentation on the dorsum of the tongue.

During the clinical examination, the patient displayed widespread reticulate hyperpigmentation on the trunk, face, neck, upper limbs, palms and soles (Fig 1). The areolae also showed increased pigmentation (Fig 2). Examination of the scalp showed diffuse, nonscarring hair loss in the frontal, temporal, parietal, and occipital areas (Fig 3). The hair-pull test yielded negative results. All fingernails exhibited onychodystrophy, with some toenails also being affected (Fig 4). Dermatoglyphics were absent (Fig 5). The oral examination revealed mucosal lesions and diffuse hyperpigmentation on the dorsum of the tongue (Fig 6), normal teeth and no signs of pallor. Physical and mental examinations were both normal.

**Fig 7:**
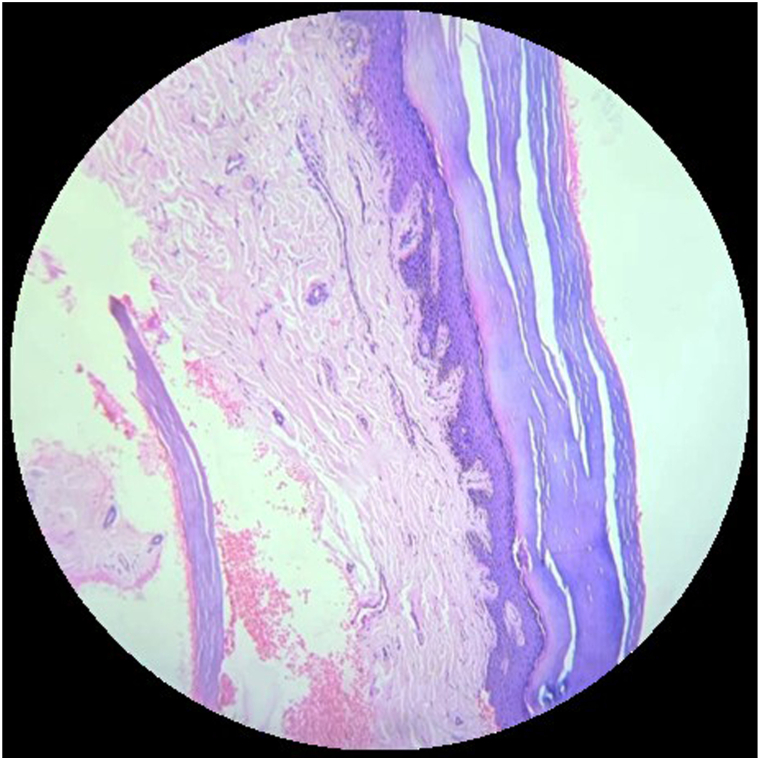
Punch biopsy of a pigmented lesion on the trunk showing compact hyperkeratosis, irregular bulbous acanthosis, basal layer hyperpigmentation, and mild upper dermal lymphocytic infiltrate with dilated capillaries.

Routine laboratory tests, such as a complete blood count, liver and kidney function assessments, and tumour markers, were all within expected parameters. Punch biopsy showed compact hyperkeratosis, irregular bulbous acanthosis and increased basal layer pigmentation. Upper dermis showed dilated congested capillaries with mild lymphocytic infiltrate; deep dermis was normal (Fig 7).

## Case 2

On extended history taking, it was noted that, the 28 y old sister of case 1, born of a non-consanguineous marriage, also presented to the outpatient dermatology department with similar symptoms including reticulate hyperpigmentation all over the body and nail dystrophy since birth, along with hypohidrosis and heat intolerance that worsens during summer. The patient reported no fading or reduction of pigmentation since its onset in early childhood, and no childhood photographs were available for retrospective comparison. Notably, there were no other affected first- or second-degree relatives. On general physical examination, the patient appeared thin with xerotic skin, while systemic, scalp and mental examinations were unremarkable. Baseline investigations were within normal limits.

**Fig 8:**
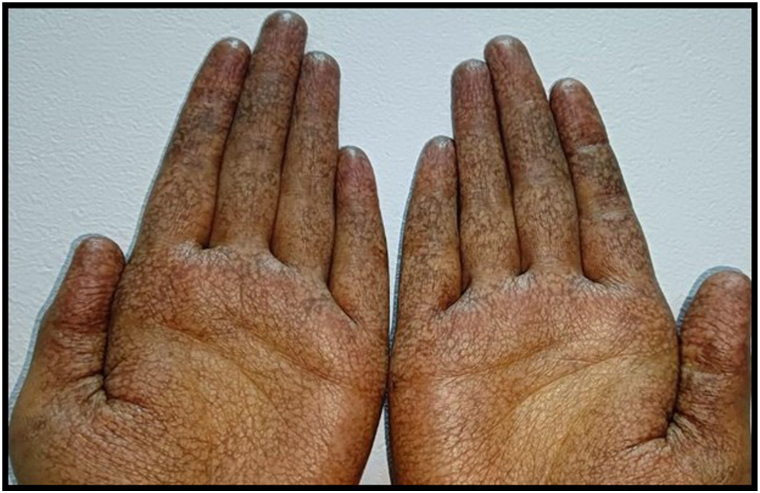
Reticulate hyperpigmentation of palms.

**Fig 9:**
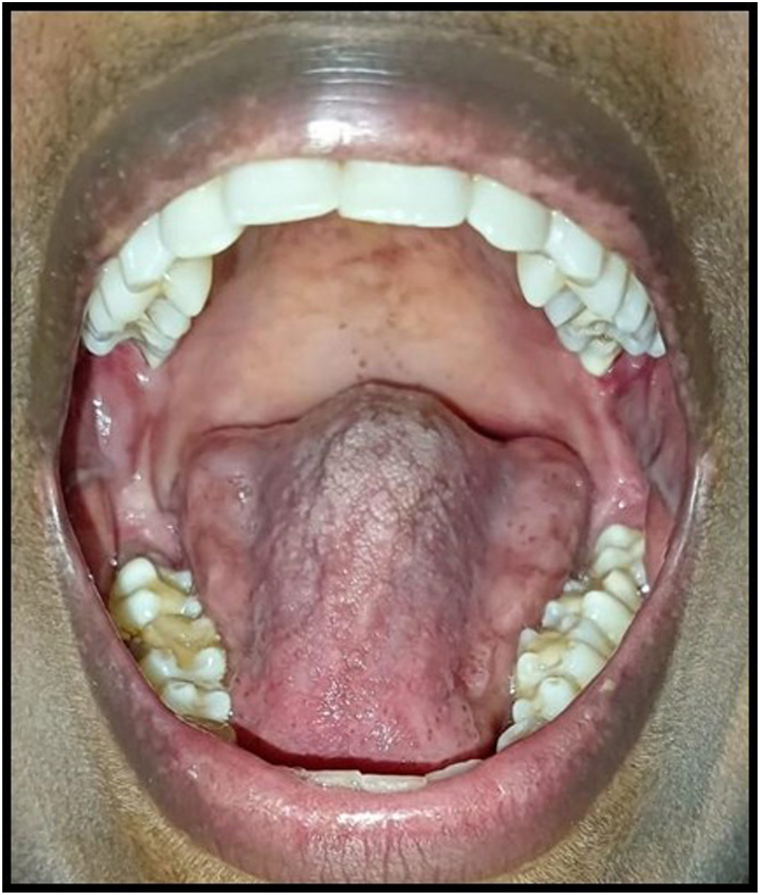
Abnormal dentition with yellow discoloration and enamel defects along with mucosal lesions on the tongue.

**Fig 10:**
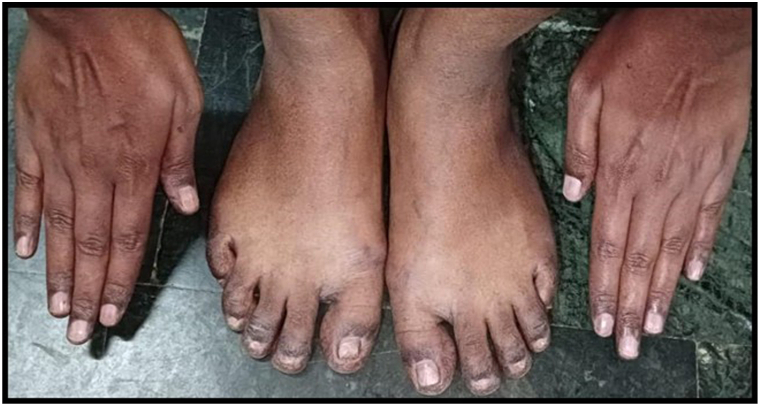
Onychodystrophy and brown coloration fingers and toenails along with xerotic and shiny skin over the dorsal aspect of hands and feet.

**Fig 11:**
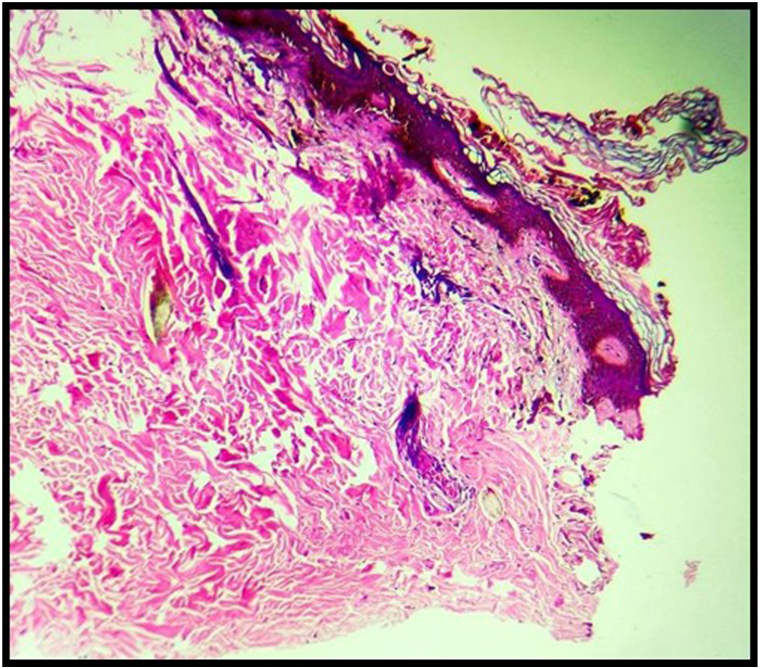
Biopsy of hyperpigmented macule on the back showing hyperkeratosis with focal pigment in the keratin layer, increased melanin pigmentation of the basilar keratinocytes with increased number of melanocytes, scattered dermal melanophages and minimal pericapillary inflammation.

On dermatological examination, the patient had reticulate pigmentation over the entire body, including the palms and soles (Fig 8). On oral examination, mucosal lesions were present on the tongue as well as abnormal dentition with yellow discoloration and enamel defects (Fig 9). The skin on the dorsal aspects of the hands and feet appeared to be xerotic and shiny, whereas the finger- and toenails were severely dystrophic (Fig 10), with an absence of dermatoglyphics. Biopsy from a hyper pigmented macule showed hyperkeratosis with focal pigment in the keratin layer, increased melanin in basal keratinocytes, increased melanocytes, scattered dermal melanophages and minimal pericapillary inflammation (Fig 11). Molecular analysis involving a dysplasia gene panel and Epidermolysis Bullosa (EB) genepanel was also used to rule out overlap with EB.This case report was prepared in accordance with the CARE (Case Report) guidelines.^4^

## Discussion

NFJS and DPR are autosomal dominant ectodermal dysplasias with overlapping phenotypic characteristics such as underdeveloped dermatoglyphics, reticulate hyperpigmentation of the skin, hypohidrosis, and heat intolerance, all of which are present in both our cases. Furthermore, DPR is distinguished by a triad of generalized reticulate hyperpigmentation, nonscarring alopecia, and onychodystrophy.^1^^,^^5^ The triad mentioned above is present in the male patient, making a clear diagnosis of DPR. Both NFJS and DPR are currently frequently regarded as distinct types of the same condition because of their comparable clinical presentations and shared gene alterations (Table I).^6^

**Table I:** Comparison of NFJS vs DPR

Feature	NFJS	DPR	Explanation
Inheritance pattern	Autosomal dominant	Autosomal dominant	Both are inherited in an autosomal dominant pattern and classified under ectodermal dysplasias.
Gene involved	KRT14 (non-helical E1/V1 domain mutation)	KRT14 (non-helical E1/V1 domain mutation)	Both syndromes are caused by mutations in the same gene, affecting keratin 14, essential for skin, hair, and sweat glands.
Phenotypic overlap	Yes	Yes	Both show overlapping features like adermatoglyphia, hypohidrosis, and reticulate hyperpigmentation.
Distribution of Pigmentation	Trunk, proximal limbs, flexures, periocular/oral areas, palms and soles	Truncal predominance	Pigmentation mostly affects the trunk, flexures, and periocular/oral areas in NFJS and fades after puberty. In DPR, it has a truncal predominance, persistent throughout life.
Alopecia	Absent	Diffuse, nonscarring alopecia present	Alopecia is a hallmark of DPR and not seen in NFJS, useful for clinical differentiation.
Nail dystrophy	Present	Uncommon	Both may have onychodystrophy, but it is part of the classical triad in DPR.
Enamel defects	Present; often leads to premature complete loss of teeth.	Not typical	Enamel defects are a diagnostic clue for NFJS; rare in DPR.
Dermatoglyphics	Absent (adermatoglyphia)	Absent (adermatoglyphia)	Poorly developed dermatoglyphics are shared features.
Course of pigmentation	Improves in adulthood	Lifelong persistence	Only NFJS pigmentation fades, which helps in retrospective diagnosis.
Management	Supportive care, heat protection, sufficient hydration, oral antioxidants, and topical emollients. Avoid strenuous physical activities	Supportive care, heat protection, sufficient hydration, oral antioxidants, and topical emollients. Avoid strenuous physical activities	No definitive cure exists for either. Genetic counseling is recommended for individuals considering parenthood.

NFJS and DPR have been linked with mutations in the non-helical E1/V1 domains of KRT14. This gene is crucial for synthesizing keratin 14, a protein integral to the structure and function of the skin, hair, and sweat glands. A deficit in keratin 14 elevates the probability of epidermal cells going through apoptosis, resulting in the skin and nail anomalies characteristic of NFJS/DPR.^7^ The key symptoms and signs of NFJ syndrome, as identified by Franceschetti and Jadassohn, include reticulate pigmentation.^8^ This pigmentation ranges from brown to gray-brown and is localized on the trunk, proximal extremities, axillae, groins, flexures, and periocular and perioral areas, with a gradual fading beginning around the age of 15 years.^1^ Furthermore, this hyperpigmentation can also be evident on the palmar and plantar surfaces, including the periungual regions of the fingers and toes, as demonstrated in the female patient's instance, where the reticulate hyperpigmentation is clearly visible over the palms and soles.^7^ Importantly, while NFJS classically exhibits post-pubertal fading of reticulate hyperpigmentation, documented exceptions exist—some patients retain pigment into adulthood, reflecting phenotypic variability within the syndrome.^8^ While in DPR, reticular pigmentation with a truncal preponderance appears in infancy or early childhood and remains throughout life.^1^^,^^5^ In our male patient, diffuse brown hyperpigmentation was present initially on the trunk at birth that later spread to the face, neck, proximal extremities, palms, soles, and tongue, all within 3 years, and he also had increased pigmentation of the areolae. The female patient mentioned above has all the clinical features of the NFJS, which are mentioned in the literature, but her pigmentation is persistent to date without fading, which is more in favor of DPR. Hence, we were compelled to make a diagnosis of overlap syndrome in our patient. Therefore, the most notable distinction between these 2 conditions is that in NFJ syndrome, pigmentation consistently improves in adulthood.^8^

Although palmoplantar keratoderma, nail dystrophy, and enamel abnormalities that often result in significant dental impairment and premature complete loss of teeth are often prevalent in NFJS, the presence of diffuse noncicatricial alopecia is a differentiating characteristic exclusive to DPR. While noncicatricial alopecia corresponds to the presentation of our male patient, it is prudent to think about the diagnosis of DPR in any child exhibiting diffuse reticulate hyperpigmentation that commences in early childhood, as it may be the sole manifestation of DPR prior to puberty, even in the absence of alopecia and onychodystrophy.^5^^,^^9^

Like NFJS and DPR, Dyskeratosis Congenita (DKC) is another form of inherited reticulate pigmentary disorder, which is characterized by a triad of congenital reticulate hyperpigmentation, especially on the neck and chest, with leukoplakia and nail dystrophy in fingernails and toenails. Apart from the cutaneous features, it is also characterized by hematological abnormalities like bone marrow failure, predisposition to malignancies, liver failures, and pulmonary complications, none of which is present in both our patients even though they share the features of the triad mentioned above.^3^

Similar to other ectodermal dysplasias, patients with DPR and NFJS should restrict heat exposure and ensure sufficient hydration^8^ along with oral antioxidants and topical emollients that can improve skin health, and patients should refrain from strenuous physical activity.^7^ Symptomatic management and genetic counseling are advised for afflicted individuals contemplating parenthood.^8^

Our cases contribute to the further understanding of this rare condition. In addition, to the best of our knowledge, overlap syndrome and DPR in siblings have not been previously reported in literature.

### Declaration of generative AI and AI-assisted technologies in the writing process

The comparison table in the Discussion was formatted with the assistance of an AI tool (ChatGPT) for structural clarity. The clinical and literature-based content was author-generated and reviewed.

## Conflicts of interest

None disclosed.
